# Postpartum OGTT Non-Adherence in Regional and Rural Australia: A Longitudinal Study

**DOI:** 10.3390/ijerph23040539

**Published:** 2026-04-21

**Authors:** Michelle Culhane, Shelley Jedrisko, Joanne Harris, Michelle Johnson, Nourah Lababidi, Christina Aggar

**Affiliations:** 1 Northern NSW Local Health District (NNSWLHD), Lismore, NSW 2480, Australia; shelley.jedrisko@health.nsw.gov.au (S.J.); joanne.harris2@health.nsw.gov.au (J.H.); michelle.johnson6@health.nsw.gov.au (M.J.); 2 Faculty of Health, Southern Cross University, Lismore, NSW 2490, Australia; nourah.lababidi@scu.edu.au (N.L.); christina.aggar@scu.edu.au (C.A.)

**Keywords:** gestational diabetes mellitus (GDM), postpartum, oral glucose tolerance test (OGTT)

## Abstract

**Highlights:**

**Public health relevance—How does this work relate to a public health issue?**

- Postpartum OGTT screening after GDM is critical for early detection and prevention of type 2 diabetes, yet uptake remains low, particularly in regional and rural settings.
- This study examines lifestyle behaviours and health-related quality-of-life factors associated with OGTT non-adherence over time.

**Public health significance—Why is this work of significance to public health?**

- OGTT non-adherence increased across the postpartum period, indicating declining engagement over time.
- No consistent predictors were identified, suggesting all women with prior GDM remain at risk of missed follow-up.

**Public health implications—What are the key implications or messages for practitioners, policy makers and/or researchers in public health?**

- Early postpartum represents an important opportunity to support screening and prevention behaviours.
- Long-term engagement may require flexible, integrated, and accessible follow-up models, particularly for regional and rural populations.

**Abstract:**

**Background:** Postpartum oral glucose tolerance test (OGTT) screening after gestational diabetes mellitus (GDM) enables early detection and prevention of type 2 diabetes, yet adherence is suboptimal, particularly in regional and rural areas. This study examined lifestyle behaviour and health-related quality-of-life factors associated with OGTT non-adherence over time. **Methods:** A longitudinal cohort study of women with prior GDM in regional and rural New South Wales, Australia, was conducted. Binary logistic regression models examined associations between lifestyle behaviours, health-related quality of life, and OGTT non-adherence at 3, 18, and 36 months postpartum. **Results:** OGTT non-adherence increased over time. Multivariable models were not statistically significant at any timepoint. At 3 months postpartum, several lifestyle and health-related quality-of-life variables were associated with non-adherence; however, these associations were not sustained at later timepoints. No consistent predictors of non-adherence were identified across follow-up. **Conclusions:** All women with prior GDM remain at risk of missed postpartum screening, with engagement declining over time. Findings should be interpreted as exploratory, reflecting time-specific patterns rather than stable predictors. Early postpartum represents a critical window for intervention, while longer-term strategies require flexible, integrated, and accessible models of care to support sustained diabetes prevention, particularly in regional and rural populations.

## 1. Introduction

Gestational diabetes mellitus (GDM) is a common pregnancy- complication and a strong predictor of future type 2 diabetes mellitus (T2DM), with up to 65% of affected women developing T2DM within 5–16 years postpartum [1]. Exposure to intrauterine hyperglycaemia also increases long-term metabolic risk for children, including obesity, impaired glucose tolerance, and T2DM, reinforcing the intergenerational public health significance of GDM [2,3]. The prevalence of GDM continues to rise globally [4]. In Australia, approximately 18% of pregnancies are affected by GDM, placing increasing demand on maternity, primary care, and chronic disease prevention services [5].

From a public health and population surveillance perspective, the postpartum period following GDM represents a critical opportunity to identify ongoing dysglycaemia and intervene early to reduce future T2DM risk. Clinical guidelines from the Australasian Diabetes in Pregnancy Society and the Royal Australian College of General Practitioners recommend postpartum oral glucose tolerance testing (OGTT) within 6–12 weeks of birth to detect persistent glucose intolerance and guide prevention strategies [6]. Despite these recommendations, non-adherence to postpartum OGTT screening remains consistently high in Australia, with particularly poor uptake reported among women living in regional, rural, and remote areas [7,8,9,10]. International recommendations similarly identify low postpartum screening rates as a critical gap in diabetes prevention pathways and call for strengthened early follow-up after GDM [11].

Low postpartum OGTT uptake reflects a complex interplay of individual and health system factors. Individual-level barriers include competing postpartum demands, communication challenges, cultural differences, limited awareness of future diabetes risk, fear of diagnosis, and logistical challenges such as transport and financial constraints [12,13,14]. Health system barriers, including fragmented transitions between maternity services and primary care, inconsistent recall and reminder systems, and limited integration of postpartum diabetes pathways, further undermine continuity of care and screening participation [15,16]. These challenges are amplified in regional and rural settings, where access to pathology services and preventive health infrastructure is often limited [9].

Lifestyle behaviours are increasingly recognised as important predictors of women’s engagement with preventive health services and chronic disease surveillance during the postpartum period [14,17]. Smoking and socioeconomic disadvantage are associated with poorer diabetes outcomes, while breastfeeding has protective metabolic effects for women with prior GDM [18,19,20]. More broadly, the ability to engage in recommended follow-up is influenced by women’s capacity to maintain health-promoting behaviours during early motherhood. Women’s perceived physical, psychological, and social functioning influences their ability to engage in health-related activities and prioritise preventive care [21,22]. Health-related quality of life provides an indicator of women’s ability to attend to their own health while managing the competing demands of infant care and other responsibilities [22,23].

Despite increasing recognition that lifestyle behavioural factors and health-related quality of life influence postpartum care, there is limited population-level evidence on their association with OGTT non-adherence, especially in regional and rural populations. Understanding these predictors is essential for informing targeted, equity-oriented public health strategies that strengthen postpartum surveillance and diabetes prevention pathways.

This study aimed to examine lifestyle behaviour and health-related quality-of-life factors associated with non-adherence to postpartum OGTT screening among women diagnosed with GDM in regional/rural NSW Australia.

## 2. Methods

### 2.1. Study Design

A longitudinal study design was employed, with data collected at three postpartum timepoints, 3, 18, and 36 months, corresponding to recommended periods for diabetes screening following a GDM diagnosis.

### 2.2. Participants and Procedure

Eligible women included those who were 18 years of age or older and had a diagnosis of GDM in their pregnancy. Exclusion criteria were women with pre-existing type 1 or type 2 diabetes and women who experienced a traumatic birth or significant neonatal complications. Women diagnosed with GDM and referred to the Diabetes Service, were informed about the study and invited to participate. Those women indicating an interest in the study were asked to complete a consent form providing permission for the study investigators to contact them at 3, 18 and 36 months after their expected due date.

### 2.3. Ethics

Ethical approval for this study was obtained from the North Coast NSW Human Research Ethics Committee (Approval No. LNR217 2020/ETH01806). The study was conducted in accordance with the Declaration of Helsinki. All participants provided written informed consent prior to participation.

### 2.4. Measures

OGTT adherence was assessed via participant self-report at each timepoint. Participants were asked whether they had completed recommended postpartum glucose testing within the specified follow-up period. This approach may be subject to recall and social desirability bias, particularly at later timepoints. Lifestyle behaviours included smoking status, breastfeeding status, repeat pregnancy, physical activity, and nutrition. Physical activity and dietary intake were assessed against the Australian national guideline recommendations [5]. Health-related quality of life was assessed using the 36-Item Short Form Health Survey (SF-36) [24]. The SF-36 evaluates multiple dimensions of wellbeing, including general health perceptions, physical functioning, role limitations due to physical or emotional health problems, social functioning, emotional wellbeing, energy and fatigue levels, and bodily pain. These domains provide a comprehensive understanding of how individuals perceive their overall health status and the extent to which physical and emotional factors impact their daily lives.

### 2.5. Data and Analysis

Data were screened prior to analysis. Participants with complete missing data across outcome and predictor variables were excluded at each timepoint (3 months: *n* = 31; 18 months: *n* = 10; 36 months: *n* = 11). Missing data were assessed using Little’s MCAR test; however, given its limited power in smaller samples, results were interpreted cautiously. Multiple imputation was applied at the 3-month timepoint where missingness exceeded 20%, while expectation–maximisation imputation was used at later timepoints where missingness was lower. This approach was pragmatic and guided by the extent and pattern of missing data rather than a strict threshold.

Sample size adequacy for logistic regression analyses was assessed using the commonly recommended guideline of a minimum of 10 participants per predictor variable [25].

Binary logistic regression models were fitted at each timepoint to examine lifestyle behaviour and health-related quality-of-life factors associated with OGTT non-adherence. The outcome was coded as non-adherence = 1 and adherence = 0. Odds ratios (ORs) > 1 indicate higher odds of non-adherence, whereas ORs < 1 indicate lower odds of non-adherence.

Separate multivariable models were fitted for: (1) lifestyle behaviour predictors; and (2) health-related quality-of-life predictors. SF-36 domains were scored and reverse-coded where required. Raw item scores were transformed to a 0–100 scale using recommended algorithms, with higher scores indicating better perceived health status [24]. All predictors were assessed for normality and multicollinearity prior to inclusion in regression models. Effect estimates are reported as odds ratios (ORs) with 95% confidence intervals, and statistical significance was set at *p* < 0.05.

Separate multivariable models were specified for lifestyle behavioural and health-related quality-of-life factors to address conceptual, statistical, and interpretive considerations. First, lifestyle behaviour factors (e.g., physical activity and dietary) represent modifiable health behaviours that may directly influence engagement with preventive screening, including non-adherence to recommended OGTT testing [14,17]. In contrast, health-related quality-of-life factors capture broader perceptions of physical, emotional, and social functioning, which may influence screening non-adherence indirectly by shaping health priorities, perceived capacity, and competing demands [21,22,23]. Modelling these predictor sets separately allowed for clearer examination of their distinct theoretical contributions to OGTT non-adherence. Second, the SF-36 comprises multiple interrelated subscales that are moderately to highly correlated. Including all SF-36 domains alongside lifestyle behavioural predictors within a single model would increase the risk of multicollinearity and model overfitting, particularly given sample size constraints at each timepoint. Separate models therefore improved model stability and reduced the likelihood of inflated standard errors.

Finally, the use of separate models enhanced interpretability by enabling direct comparison of predictors within conceptually coherent domains, while maintaining adequate statistical power and meeting recommended events-per-variable guidelines for logistic regression. The number of events (non-adherence cases) and predictors included at each timepoint were considered in relation to recommended events-per-variable guidelines; however, these assumptions may not have been fully met at later timepoints due to reduced sample size. Variable selection was informed by existing literature and clinical relevance, with the aim of capturing behavioural and health-related factors associated with engagement in postpartum care.

## 3. Results

A total of 121 participants completed Timepoint 1 (T1), 70 completed Timepoint 2 (T2), and 83 completed Timepoint 3 (T3). The descriptive analysis in Table 1 shows an increase in non-adherence to postpartum OGTT screening over time, with 61.2% of participants completing the test at T1, 42.9% at T2 and 48.2% at T3. Similarly, as expected, breastfeeding rates decreased significantly from 70.2% at T1 to 30.0% at T2, and 9.3% at T3. Smoking rates also showed a downward trend, with only 1.6% of participants reporting smoking at T3 compared to 9.9% at T1 (Table 1).

Given the reduced sample size at later timepoints, estimates may be less stable and confidence intervals wider, which should be considered when interpreting associations across time. The overall multivariable logistic regression models were not statistically significant at any timepoint. Accordingly, results are presented as exploratory analyses, and individual associations should be interpreted with caution. Given the number of predictors examined across multiple timepoints and model sets, there is an increased risk of type I error.

Although the models were not statistically significant, several individual variables showed associations with OGTT non-adherence at specific timepoints (Table 2). These findings are reported to describe potential time-dependent patterns rather than independent predictors.

**Lifestyle behaviour**

At T1, several lifestyle factors showed associations with OGTT non-adherence. Higher body mass index (OR = 0.991, *p* = 0.001), meeting physical activity guidelines (OR = 0.870, *p* = 0.019) and dietary recommendations (OR = 0.606, *p* < 0.001) were associated with lower odds of OGTT non-adherence (i.e., reduced likelihood of missing the OGTT). At T2 and T3, no lifestyle behaviour factors were significantly associated with OGTT non-adherence. These findings suggest that any observed associations in early postpartum were not sustained over time. Physical activity was excluded from the T2 model due to sample size constraints, and BMI was excluded from the T3 model due to a highly skewed distribution that violated regression assumptions. No lifestyle behaviour factors demonstrated consistent associations with OGTT non-adherence across all timepoints.

**Health-related quality of life**

At T1, several health-related quality-of-life factors were associated with OGTT non-adherence. Higher scores for General Health (OR = 0.989, *p* = 0.002), Social Functioning (OR = 0.987, *p* < 0.001), and Energy/Fatigue (OR = 0.993, *p* = 0.045) were associated with lower odds of non-adherence. In contrast, higher Role Emotional scores were associated with higher odds of non-adherence (OR = 1.011, *p* = 0.002). At T2, no health-related quality-of-life factors were significantly associated with OGTT non-adherence. At T3, Social Functioning was significantly associated with higher odds of OGTT non-adherence (OR = 1.045, *p* = 0.037). Several health-related quality-of-life factors, including Physical Functioning and Role Physical, were excluded from selected models due to skewed distributions or violations of regression assumptions, to preserve model validity and reduce bias.

Overall, associations between predictors and OGTT non-adherence varied across timepoints and should be interpreted as exploratory, time-specific signals rather than stable or independent predictors.

## 4. Discussion

This longitudinal study examined lifestyle behaviour and health-related quality-of-life factors associated with non-adherence to postpartum OGTT screening among women diagnosed with GDM in regional and rural NSW Australia. All associations described should be interpreted as exploratory signals rather than explanatory or causal relationships, particularly given the absence of statistically significant multivariable models and the likelihood that events-per-variable assumptions were not met at later follow-up points.

The multivariable models were not statistically significant at any timepoint, indicating that the included variables did not collectively explain variation in OGTT non-adherence. This finding is consistent with previous longitudinal studies that have not identified stable predictors of postpartum screening, suggesting that all women with prior GDM may remain at risk of missed follow-up [7,26,27,28]. Accordingly, the associations observed at individual timepoints should be interpreted as exploratory and hypothesis-generating, rather than indicative of independent or causal relationships.

Non-adherence increased over time, demonstrating a sustained decline in engagement [17,22,28]. From a population health perspective, this pattern highlights an opportunity for early identification and prevention of type 2 diabetes mellitus (T2DM) among a high-risk group, with important implications for long-term chronic disease burden and health system costs [27,28,29,30,31,32].

At three months postpartum, several lifestyle and health-related quality-of-life variables were associated with OGTT non-adherence; however, these findings should be interpreted cautiously given the lack of overall model significance. These patterns may represent exploratory signals that engagement in health-promoting behaviours and perceived wellbeing could be associated with perceived capacity to engage with preventive care during early motherhood. While these observations are consistent with behavioural health models that link health behaviours to engagement with preventive services [28,33], they should not be interpreted as evidence of independent predictors. Similarly, associations have been reported in women with prior GDM, particularly in the early postpartum period when perceived risk and contact with health services remain relatively high [21,30,34]. The association between higher BMI and lower non-adherence at three months postpartum was counterintuitive. One speculative explanation is there is greater perceived diabetes risk among women with higher BMI [27,35]; however, alternative explanations such as increased clinical monitoring or provider-driven follow-up should also be considered. Given the exploratory nature of the findings, this result requires cautious interpretation and further investigation. Higher Role Emotional scores were associated with increased odds of non-adherence; however, this should be interpreted as a potential signal rather than a definitive relationship. Role limitations due to emotional problems may reflect psychological distress, emotional exhaustion, or difficulty balancing competing roles during early motherhood, all of which have been shown to impede engagement with preventive healthcare [23].

Higher social functioning showed time-dependent associations with OGTT non-adherence. Importantly, the direction of this association differed across timepoints, reinforcing the absence of stable predictors and suggesting that relationships between wellbeing and preventive care engagement may change over time. At 3 months, higher social functioning was associated with lower odds of OGTT non-adherence, which may be consistent with the hypothesis that social support could facilitate early engagement; however, this interpretation remains speculative [36]. There is growing evidence that women supported by partners and family members are more likely to engage in postpartum care [21,37]. By 36 months higher social functioning was associated with higher odds of OGTT non-adherence, which may reflect competing demands such as employment, caregiving, and community roles as time from pregnancy increases [22,23,28]. This changing balance between capacity and competing demands has important implications for equity-oriented service delivery in regional and rural area, where travel, cost, and limited service availability already constrain access [7,9]. Early strategies should therefore leverage family, partner, and community support and existing maternity and child and family health contacts to facilitate screening, while longer-term approaches require flexible, low-burden models embedded in primary care and community settings—such as coordinated recall systems, opportunistic testing, and digitally supported reminders—to align with women’s changing life circumstances and reduce geographic inequities in follow-up. These interpretations remain speculative and should be considered in light of the exploratory nature of the findings.

The absence of statistically significant multivariable models, combined with variation in associations across timepoints, indicates that OGTT non-adherence is likely influenced by complex, multifactorial, and context-dependent factors that were not fully captured in this study. These findings reinforce the limitations of risk-based approaches and highlight the need for system-level strategies that support all women with prior GDM [28].

### 4.1. Implications for Practice, Policy, and Future Research

These findings suggest that improving postpartum OGTT adherence requires system-level, rather than risk-based, approaches. Given the lack of stable predictors, interventions should not rely on individual risk profiling but instead prioritise universal, accessible, and sustained follow-up strategies. In practice, these findings suggest that screening may benefit from being initiated early and embedded within routine maternity, child and family health, and primary-care contacts, with flexible testing options and involvement of partners or family to help reduce logistical and social barriers. Policy approaches may consider normalising postpartum diabetes surveillance as a standard component of ongoing women’s healthcare, supported by coordinated recall, opportunistic testing, and digital reminders to address rural access inequities. Future research should prioritise evaluation of equity-oriented, integrated models of care—particularly early postpartum testing and primary-care-led pathways—and examine their impact on long-term engagement and T2DM prevention.

### 4.2. Strengths and Limitations

Strengths of this study include its longitudinal design with follow-up extending to 36 months postpartum and its focus on a regional and rural population, which is underrepresented in the literature. Limitations include reliance on self-reported lifestyle behavioural measures, exclusion of structural and health system variables, and reduced sample size at later follow-up points, which may have limited statistical power. Substantial attrition across timepoints may have introduced selection bias and reduced the stability of regression estimates.

Additionally, temporal misclassification may have occurred, as delayed initial testing could not be distinguished from repeated recommended testing, which may bias longitudinal interpretation of testing patterns.

The use of multiple models and repeated testing across timepoints increases the risk of type I error. While imputation methods were applied, assumptions regarding missing data cannot be fully verified. Accordingly, findings should be interpreted with caution.

## 5. Conclusions

In this longitudinal study of women with prior GDM in regional and rural Australia, postpartum OGTT non-adherence increased over time with no consistent predictors identified. The absence of statistically significant multivariable models and variability across timepoints suggest that non-adherence is influenced by complex, context-dependent factors rather than stable individual characteristics.

These findings indicate that all women with prior GDM remain at ongoing risk of missed follow-up, supporting the need for universal, system-level approaches to postpartum diabetes surveillance. Early postpartum represents a key window for intervention, while sustained engagement requires flexible, accessible models of care that address structural barriers and support long-term diabetes prevention.

## Figures and Tables

**Table 1:** Descriptive characteristics and OGTT non-adherence across postpartum timepoints (T1–T3).

Variable	T1 (%)	T2 (%)	T3 (%)
OGTT Taken	Yes: 74 (61.2%) No: 47 (38.8%)	Yes: 30 (42.9%) No: 40 (57.1%)	Yes: 40 (48.2%) No: 43 (51.8%
Smoking Status	Yes: 12 (9.9%) No: 109 (90.1%)	Yes: 7 (10%) No: 63 (90%)	Yes: 1 (1.2%) No: 82 (98.8%)
Currently Breastfeeding	Yes: 85 (70.2%) No: 36 (29.8%)	Yes: 21 (30%) No: 49 (70%)	Yes: 8 (9.6%) No: 75 (90.4%)
Repeat Pregnancy	-	Yes: 16 (22.9%) No: 54 (77.1%)	Yes: 3 (3.6%) No: 80 (96.4%)

**Table 2:** Behavioural and lifestyle factors, and health-related quality-of-life domains and OGTT non-adherence over three timepoints.

Variables	OR (95% CI) *p* Value (T1)	OR (95% CI) *p* Value (T2)	OR (95% CI) *p* Value (T3)
Smoking Status	0.944 (0.590–1.511), *p* = 0.811		1.016 (0.225–4.594) *p* = 0.984
Currently Breastfeeding	1.126 (0.831–1.526), *p* = 0.444	1.258 (0.406–3.904), *p* = 0.691	0.710 (0.163–3.097), *p* = 0.649
Repeat Pregnancy		1.739 (0.534–5.657), *p* = 0.358	0.654, (0.211–2.027) *p* = 0.462
BMI	0.991 (0.985–0.996) *p* <0. 001	0.960 (0.884–1.043) *p* = 0.337	
Physical Activity	0.870 (0.773–0.977) *p* = 0.019		1.067 (0.692–1.645) *p* = 0.770
Dietary Intake	0.606 (0.514–0.713). *p* < 0.001	0.644, (0.318–1.304) *p* = 0.221	0.649 (0.351–1.198), *p* = 0.167
**SF-36 Scale (Model 2)**			
General Health	0.989 (0.982–0.996) *p* = 0.002	1.019 (0.988–1.053) *p* = 0.230	0.994, (0.963–1.025) *p* = 0.684
Physical Functioning		0.934 (0.865–1.008) *p* = 0.078	0.963, (0.907–1.022) *p* = 0.210
Role Physical		1.026 (0.997–1.055) *p* = 0.080	1.003 (0.974–1.032) *p* = 0.861
Role Emotional	1.011 (1.006–1.015) *p* < 0.001	992 (0.970–1.014) *p* = 0.457	0.983 (0.964–1.002) *p* = 0.078
Social Functioning	0.987 (0.980–994) *p* < 0.001	1.008 (0.970–1.046) *p* = 0.691	1.045 (1.003–1.089) *p* = 0.037
Energy/Fatigue	993 (0.986–1.000) *p* = 0.045		
Bodily Pain	1.005 (0.999–1.011) *p* = 0.093		
Emotional Wellbeing	1.000 (0.991–1.010) *p* = 0.927		

Note: variables removed due to missing data. OGTT non-adherence was specified as the outcome variable (OR > 1 means non-adherent; OR < 1 means adherent).

## Data Availability

The data presented in this study are not publicly available due to ethical and privacy restrictions. De-identified data may be available from the corresponding author upon reasonable request and subject to institutional ethics approval.
